# The landscape of abnormal pathway activation confers COVID-19 patients' molecular sequelae earlier than clinical phenotype

**DOI:** 10.7150/thno.83405

**Published:** 2023-06-12

**Authors:** Xiuli Zhang, Yuan Sh, Jierong Dong, Zhongqing Chen, Feitong Hong

**Affiliations:** 1 CAS Key Laboratory for Biomedical Effects of Nanomaterials and Nanosafety, CAS Key Laboratory of Standardization and Measurement for Nanotechnology, CAS Center for Excellence in Nanoscience, National Center for Nanoscience and Technology of China, Beijing 100190, China.; 2 Fujian Provincial Key Laboratory of Brain Aging and Neurodegenerative Diseases, School of Basic Medical Sciences, Fujian Medical University, Fuzhou 350108, Fujian Province, China.; 3 The First Affiliated Hospital of Fujian Medical University, Fuzhou 350108, Fujian Province, China.

**Keywords:** COVID-19, Sequelae, pST-CA, Serum multi-omics, Abnormal pathway activation

## Abstract

**Rationale:** The 2019 coronavirus disease (COVID-19) pandemic poses a significant threat to human health. After SARS-CoV-2 infection, major clinical concerns are organ damage and possible sequelae.

**Methods:** In this study, we analyzed serum multi-omics data based on population-level, including healthy cohort, non-COVID-19 and COVID-19 covered different severity cohorts. We applied the pseudo-SpatioTemporal Consistency Alignment (pST-CA) strategy to correct for individualized disease course differences, and developed pseudo-deterioration timeline model and pseudo-recovery timeline model based on the "severe index" and "course index". Further, we comprehensively analyzed and discussed the dynamic damage signaling in COVID-19 deterioration and/or recovery, as well as the potential risk of sequelae.

**Results:** The deterioration and course models based on the pST-CA strategy can effectively map the activation of blood molecular signals on cellular, pathway, functional and disease phenotypes in COVID-19 deterioration and throughout the disease course. The models revealed the neurological, cardiovascular, and hepatic toxicity present in SARS-CoV-2. The abundance of differentially expressed proteins and the activity of upstream regulators were comprehensively analyzed and evaluated to predict possible target drugs for SARS-CoV-2. On molecular docking simulation analysis, it was further demonstrated that blocking CEACAM1 is a potential therapeutic target for SARS-CoV-2.

**Conclusions:** Clinically, the risk of organ failure and death in COVID-19 patients rises with increasing number of infections. Individualized sequelae prediction for patients and assessment of individualized intervenable targets and available drugs in combination with the upstream regulator analysis results are of great clinical value.

## Introduction

The rapid outbreak of the coronavirus disease 2019 (COVID-19) was a wake-up call to the global healthcare preventive treatment system. It had a serious impact on the world's medical resources and economic trade 1. COVID-19 is an infectious respiratory disease caused by severe acute respiratory syndrome coronavirus type 2 (SARS-CoV-2) 2. By October 2022, the cumulative number of patients infected with COVID-19 has exceeded 600 million worldwide, with more than 6 million deaths, having spread to more than 150 countries and territories 3.

The clinical symptoms of patients infected with SARS-CoV-2 vary greatly and are commonly divided into symptomatic or asymptomatic 4-6. Commonly, among patients who present some typical clinical symptoms, respiratory impairment and other complications are the most prominent characteristics of the disease 7. Numerous studies have demonstrated that SARS-CoV-2 infection may cause a variety of potential sequela, including impaired lung function or imaging abnormalities, cardiovascular injury, neuroinflammation and nerve damage, gastrointestinal injury, and renal injury 8-12.

The common approach to assess the impact of abnormally expressed molecules is to assess the level of significant enrichment of biological events such as pathways. However, the possible effects of differential molecules significantly enriched in particular biological events include activation, inhibition, or no effect. The combined inclusion of the up- and down-regulation levels of activating and repressing molecules allows for an accurate quantitative assessment of the activation levels of differential molecular expression profiles to biological events.

Currently, methods such as GSEA can incorporate up- and down-regulation information of genes to assess the level of activation of biological events. However, the role of repressive genes on biological events needs to be defined manually. It is difficult to obtain all suppressor-type genes affecting each biological event such as pathways from the literature. Therefore, the lack of information on suppressor-type genes limits the accurate assessment of activation levels of differential genes affecting biological events and the subsequent assessment of dynamic activation trends among groups for the same biological event. Therefore, a panoramic picture of the activation levels of biological events at each level of the organism after SARS-CoV-2 virus infection is not available. In addition, studies based on clinical data were devoted to uncovering clinical indicators related to the progression of COVID-19 13-15. However, most of the studies have not considered the impact of the length of the disease course on different patients or the alteration of organismal biological events before and after the disease progresses more severely.

In this study, we incorporated proteomic and metabolomic data from the sera of patients infected with SARS-CoV-2 using the QIAGEN IPA (Ingenuity Pathway Analysis) platform 16, which synthetically calculated the four ways in which each differentially expressed molecule is involved in a particular biological event (including up-regulation vs activation, up-regulation vs inhibition, down-regulation vs activation, down-regulation vs inhibition). In general, we used the healthy cohort as a baseline and corrected for the length of time of the severe event and the length of the total disease course in patients, respectively. We obtained significant enrichment levels (p value) and activation levels (z-score) of particular biological events under each abnormal condition. Further, we dynamically depicted the activation trends encompassing four aspects: first, whether COVID-19 was diagnosed or not, second, the different severity levels of COVID-19 patients, third, the deterioration process of COVID-19 patients (mild to severe disease), and fourth, the recovery process of COVID-19 patients (SARS-CoV-2 from positive to negative).

We are interested in abnormal activation of biological events that occur in disease groups compared to healthy individuals. What are the differences and relationships in abnormal activation events between different groups? From micro to macro, what are the trends of molecular (micro), pathway, functional, and organ (macro) toxicity changes in the same group?

By exploring the activation trends of each biological event and the clinical sequelae after recovery under spatio-temporal dynamic conditions in SARS-CoV-2 infected population, we provide important mechanistic clues and clinical drug rationale for early intervention in multiple infections, severed patients, etc.

## Methods

### Quality control of serum multi-group data

We performed data cleansing on serum multigroup data. Data cleaning aims at missing data, abnormal data, duplicate data, and meaningless data containing wrong characters. First, we calculate the missing rate of all features and then delete features whose missing rate exceeds 10%. The remaining NA values are set to 0.5 times minimum value. Manually check the duplicate data and ensure that the duplicate data is correct, then delete any of them. For continuous features, we used the Shapiro-Wilk method to test the normality of the data. If the feature corresponds to a normal distribution, we will calculate the standard deviation. Any value of a feature that exceeds three times the standard deviation is regarded as an abnormal value, and the value will be deleted. When the data is discrete or continuous data with non-normal distribution, we calculate the quarter quantile and third quartile of characteristic data, respectively. If the number exceeds 1.5 times the quartile, it will be regarded as an abnormal value, and the data will be deleted. All statistical learning methods are based on R version 4.2.1.

### Ingenuity Pathway Analysis

Ingenuity Pathway Analysis (IPA, QIAGEN, USA) is a commercial biological information platform. It is based on Ingenuity Knowledge Base's highly structured and content-rich biological and chemical discoveries. In this study, we used the “Canonical Pathway Analysis” and “Diseases and Biofunctions” modules of IPA to annotate pathway and biological functions. P-value <0.05 was considered a statistically significant threshold. Z-score greater than 0 is defined as activation, and less than 0 is defined as inhibition. The activation z-score of a hypothesis is calculated from the regulation directions and gene expression changes of the genes in the overlap of data set and hypothesis-regulated genes. It assesses whether there is a significant pattern match between predicted and observed up- and down-regulation and predicts the activation state of the regulator (z > 0: activation, z < 0: inhibition). The activation z-score is given by:



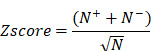



with *N+*(*N-*) being the number of genes where the product of net-effect and observed direction of gene regulation is greater (less) than zero, and *N = N+ + N-*.

### Canonical pathway analysis

We calculated the differentially expressed molecules (including proteins and metabolites) between each disease group and the healthy group. Second, we import the differentially expressed molecules between each pair of samples into the IPA (Ingenuity Pathway Analysis) analysis platform for gene annotation, canonical pathway, disease and biological function, as well as toxicity analysis. We upload the differentially expressed molecules to the IPA data catalog. Through the "Canonical Pathway Analysis" sub-module of the core analysis, we can obtain the signaling pathways significantly affected by differentially expressed molecules and their activation levels. Significance analysis for each pathway was calculated using the right-tailed Fisher's Exact Test, and -log10 (p value) > 1.3 was considered significant.

### Disease and Biological Function Analysis

The "Diseases and BioFunctions" functional analysis sub-module of the core analysis module of the IPA platform can be used to cluster the input molecules for disease and biological function analysis. The significance analysis of each disease and biological function entry was calculated using the right-tailed Fisher's Exact Test, and -log10 (p value) > 1.3 was considered significant.

### Toxicity analysis

The “Tox functions” module of IPA was used to analyze the effects of serum molecular expression profiles on the cardiotoxicity, clinical chemistry, and hematology of the organism, respectively. Significance analysis of each toxicity item was calculated using the right-tailed Fisher's Exact Test, - log10 (p value) > 1.3 was considered significant.

## Results

### Brain and nervous system function could be compromised by viral pneumonia

**Figure 1:**
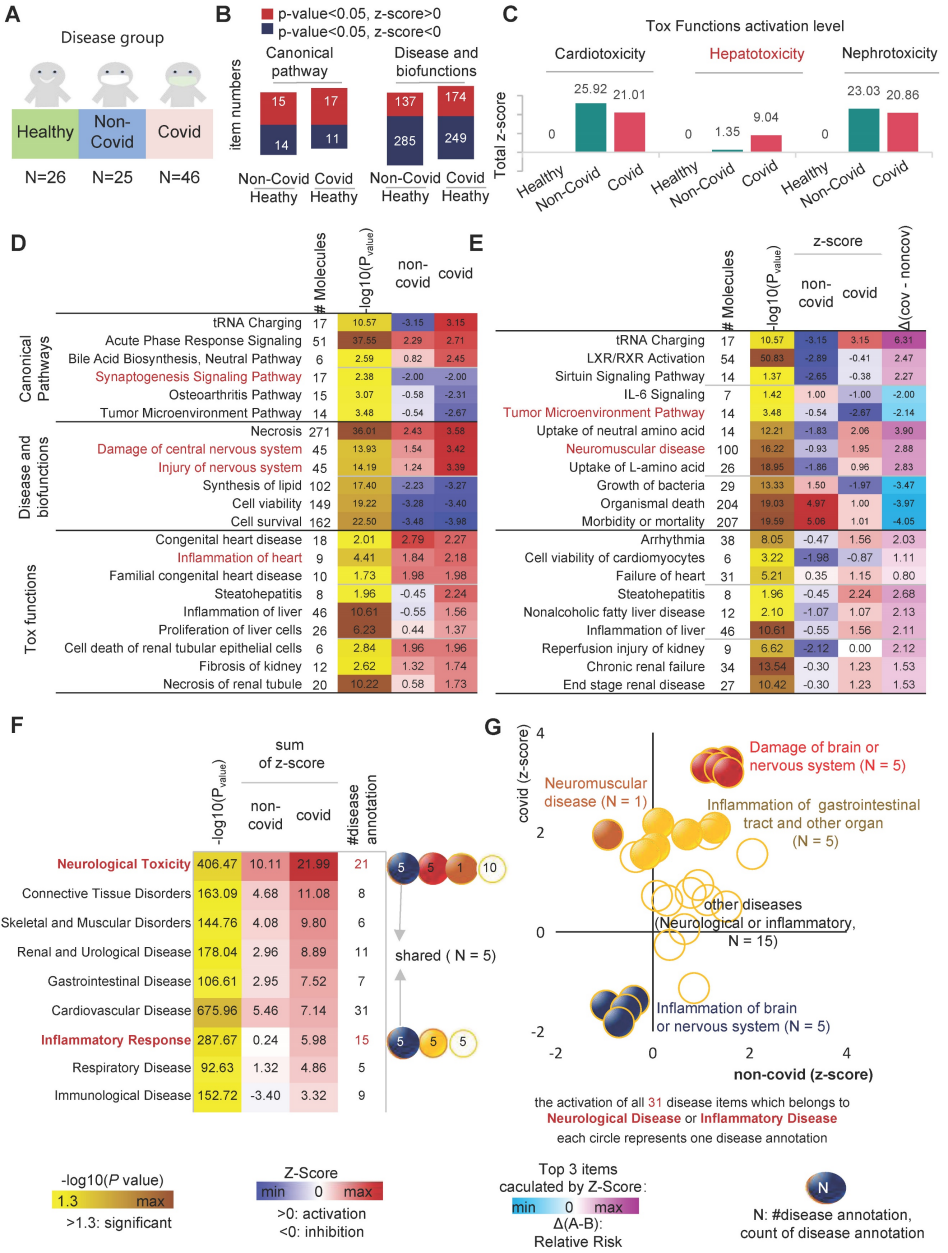
** Biological analysis of differential indicators in patients in the healthy, non-COVID and COVID groups. A**, Sample profiles of the three disease groups. **B**, Number of up- and down-regulation of differential pathways and functions in the COVID and non-COVID groups compared with the healthy group. **C**, Activation levels of cardiac, hepatic and renal toxic functions in different groups of patients compared with the healthy group. **D**, the statistically significant and interested pathways, functions and toxicity with activation (and inhibition) levels in the COVID group compared with the healthy group. **E**, the statistically significant and interested pathways, functions and toxicity with activation (and inhibition) levels in the COVID group compared with non-COVID. **F**, Diseases that were significantly activated (or inhibited). **G**, Activation level of each entry (31 items) in "Neurological Disease" (N = 21 items) and "Inflammatory Disease" (N = 15 items), where "Inflammation of brain and nervous system" (N = 5 items) was common to "Neurological Disease" (N = 21 items) and "Inflammatory Disease" (N = 15 items).

To gain a more thorough understanding of the molecular mechanisms of disease progression in COVID-19 patients. We identified similarities and differences in biological events between patients with non-COVID-19 (N = 25) and COVID-19 (N = 46), considering the healthy cohort as a control group (N = 26) (Figure 1A). To eliminate individual differences and errors between different experimental groups, if not specifically highlighted, we always used the healthy cohort as a reference baseline in this study to identify biological events that differ among all groups. There was no statistically significant difference in the number of differential canonical pathways and disease and biofunctions between patients with non-COVID-19 and COVID-19 (Figure 1B). According to the results of a toxicological functional analysis, the two distinct viral pneumonias induced differing degrees of cardiotoxicity 17,18, hepatotoxicity 19-21, and nephrotoxicity 22,23. Hepatotoxicity was greater in COVID-19 than in non-COVID-19 patients (relative risk of toxicity = 7.96, Figure 1C). Compared to the control group, both different viral pneumonias inhibited the signaling pathway of synaptogenesis. Patients with COVID-19 had more severe central nervous system (CNS) damage compared to non-COVID (Relative risk of injury = 1.88) 24-28. Cardiotoxicity caused by SARS-CoV-2 leads to severe cardiac inflammation, which may be one of the primary reasons why patients with cardiovascular disease are more likely to die from COVID-19 29,30 (Figure 1D). Although both different viral pneumonias are neurotoxic, SARS-CoV-2 has a greater capacity for neuromuscular damage (relative risk of toxicity = 2.88). In general, the degree of organ damage was more severe in COVID-19 patients compared to non-COVID-19 patients (Figure 1E). For example, neurological toxicity (relative risk = 11.88), inflammatory response (relative risk = 5.74) and gastrointestinal disorders (relative risk = 4.57).

In general, different types of viral pneumonia all cause a series of changes in biological events that lead to severe organ inflammatory responses and damage to the heart, liver, kidneys, and central nervous system and so on, resulting in compromised organ function. However, the toxicity of viruses varies, and therefore the degree of stress response produced by the organism differs (Figure 1C-D). SARS-CoV-2 infected patients have a relatively higher risk of organ and immune system damage compared to patients with non-COVID-19. CDVID-19 causes severe and distinct functional impairment, including exercise-related muscle nerve damage 31,32, metabolism-related liver toxicity, and inflammation of the gastrointestinal tract and other organs. Although both different types of viral pneumonia cause varying degrees of central nervous system (CNS) damage, CDVID-19 causes not only an associated neuroinflammatory response, but also direct damage to the brain or nervous system (Figure 1G). In this way, patients with COVID-19 have a more complex pathology of brain or neurological related diseases.

### Severe COVID-19 patients have more significant neurological or organ damage

**Figure 2:**
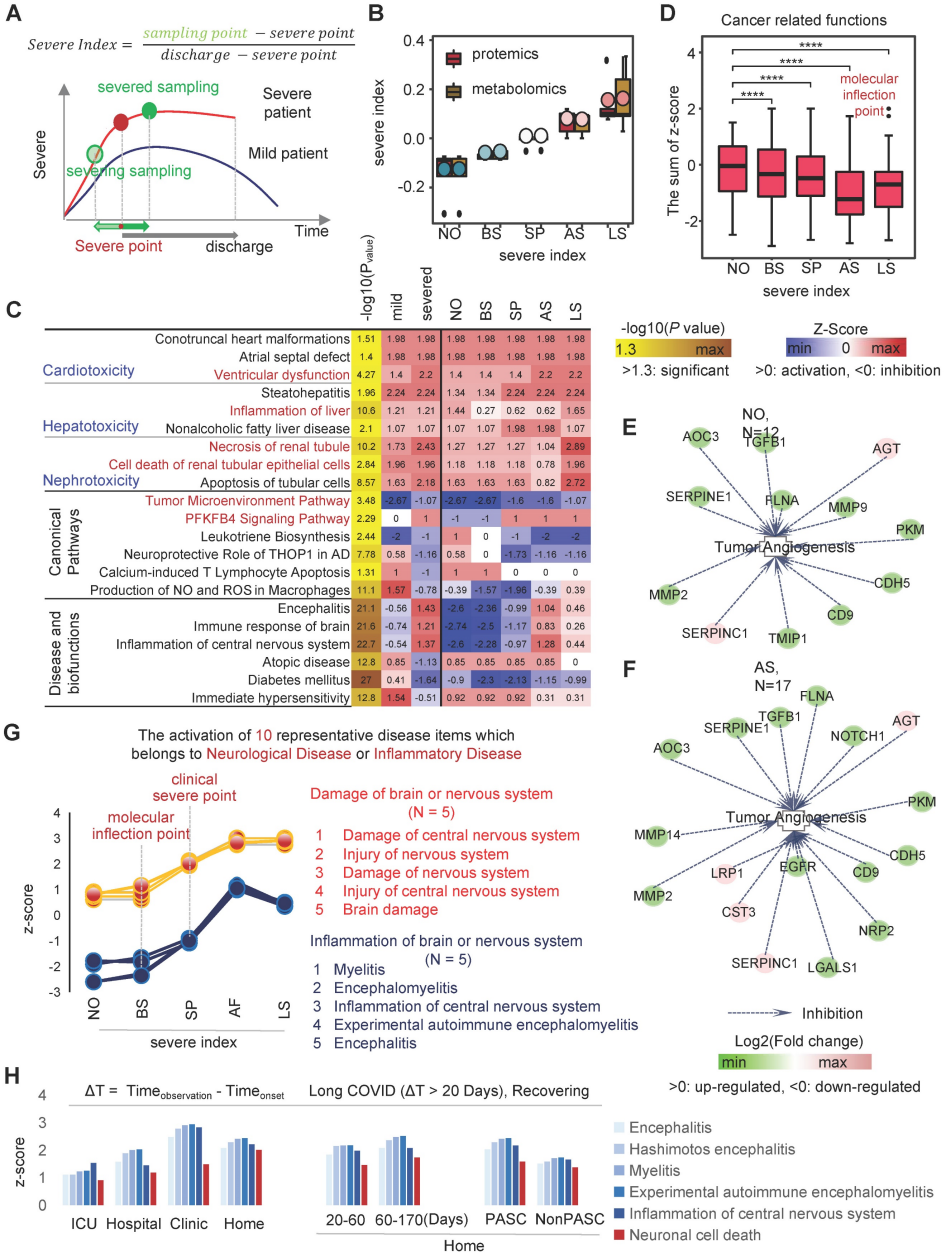
** Analysis of biological events changes in different disease status. A**, Severe index calculation diagram. **B**, Distribution of severe index for the five groups, including near onset group (NO), before-severe point group (BS), severe point group (SP), after-severe point group (AP), and persistent severe group (PS). **C**, the statistically significant and interested co-activated (and co-inhibited) canonical pathways, disease and biofunctions, as well as tox functions in both mild and severed groups. **D**, The dynamic wave of activation levels of "Cancer" related disease items (N = 98) during COVID-19 progression. **E**, NO phase, genes involved in inhibiting the tumor angiogenesis pathway. **F**, AS phase, genes involved in inhibiting the tumor angiogenesis pathway. **G**, Dynamics of neurological injury or inflammation-related biological events during the progression of COVID-19. Blue, inflammation of brain or nervous system; red, damage of brain or nervous system. **H**, As Covid-19 patients gradually recovered, the Z-score of biological events related to neurological damage in independent test sets changes. ΔT = Time_observation_ - Time_onset._

SARS-CoV-2 infected patients are primarily divided into three subgroups: asymptomatic, mild infection, and severe infection 4-6. Each of these three groups has its own immunological phenotype background and distinct clinical presentation. The severe infection is the most heterogeneous group, causing the most severe damage to the organism. To investigate the serum molecular profile of patients with different disease degrees of COVID-19, we divided the patients into a mild infection group and a severe infection group. In order to reveal the abnormal biological events occurring in the organism before and after the severe points, we corrected for the potential impact of the different duration of deterioration stages in different individuals by aligning the onset and severe points of each patient. This analysis strategy is called the pseudo-SpatioTemporal Consistency Alignment (pST-CA) strategy. By calculating the severe index for each severe patient (Figure 2A), the samples were divided into five groups according to severe index: near onset group (NO), before-severe point group (BS), severe point group (SP), after-severe point group (AP), and persistent severe group (PS) (Figure 2B).

The results of serum multi-omics data analysis showed that the toxicity of SARS-CoV-2 to organs increased gradually as the disease progressed in severe patients, resulting in different degrees of organ damage. As the disease progressed, the ventricular function deteriorated more severely, and the toxicity of SARS-CoV-2 caused different degrees of damage to the liver and kidney, resulting in increased liver inflammation acceleration of renal tubular epithelial cell death. Consequently, our evidence suggests that the organ toxicity of SARS-CoV-2 is more likely to cause sepsis and multi-organ failure during the progression of the disease in patients with severe COVID-19. In the severe infection group, CNS inflammation was more severe than in the mild infection group (Relative risk = 1.91). Notably, the immune response in the brain and the rapid deterioration of CNS inflammation occurred between BS and AS. Although it went into remission with subsequent treatment, it remained higher than SP or before (Figure 2C).

Interestingly, we found that SARS-CoV-2 affects the tumor microenvironment (TME) to some extent, leading to inhibition of tumor cell proliferation. On the timeline of COVID-19 infection, the TME inhibitory capacity of COVID-19 tended to first increase and then decrease, the inflection point occurred at AS (Figure 2C-D). For instance, in the classical tumor angiogenesis pathway, a total of 12 major marker genes operate together to inhibit tumor angiogenesis (Figure 2E). As the disease progressed to the inflection point stage, the inhibition of tumor angiogenesis by SARS-CoV-2 reached its highest level (Figure S1 A-E). A total of 17 markers were involved in co-inhibition and six markers appeared, namely MMP14, LRP1, CST3, EGFR, LGALS1, NOTCH1 and NRP2, where MMP14 replaces the pre-existing MMP9.

On the other hand, disease and functional analysis of protein and metabolic expression profiles in severe patients showed that SARS-CoV-2 caused severe damage to the CNS, especially the brain. IPA results showed that damage to the brain or neurological related signaling pathways (N=5) was continuously activated during viral infection. During the NO phase, the degree of neurological damage was low, and the slow rate of damage did not cause intense CNS inflammation. Interestingly, the inflection point event occurs in BS, when CNS inflammatory signaling pathways are highly activated and CNS damage rapidly worsens, which is one of the important presentations of severe events. When the patient progresses to the SP stage, CNS inflammation-related signaling pathways are activated at a high rate, leading to sustained CNS damage. In AS, activation of pathways associated with CNS damage and inflammatory signaling reaches its highest level (Figure 2G).

The sustained presence of neurological damage and inflammation is closely related to various post-COVID-19 sequelae, such as Anosmia/Dysgeusia, loss of taste, memory problems, anxiety, and depression. To study the relationship between neurological damage, inflammation and COVID-19 sequelae, we verified three independent individualized multi-omics data as test data 33-35. We included long COVID patients with an observation time greater than 20 days after the onset of symptoms and analyzed the samples during the recovering stage (T2/T3) 33-35. The results showed that the inflammation and "Neuronal cell death" in the nervous system remained in an activated state as patients transitioned from the peak of their severe disease (ICU) to discharge or returning home. Even when tracked up to 170 days after the onset of symptoms in discharged and returning home patients, the negative effects of SARS-CoV-2 infection on the nervous system persisted. Compared to patients with post-acute sequelae of COVID-19 (PASC), patients without sequelae (NonPASC) had slightly lower neurological damage. However, compared to healthy individuals, the neurological inflammatory damage signals of the NonPASC group still remained in an activated state (z-score > 0). This suggests that the negative, long-term, and subtle effects of SARS-CoV-2 infection on the nervous system cannot be ignored (Figure 2H).

### COVID-19 pseudo-recovery timeline model reveals changes in biological events during the course of the disease

**Figure 3:**
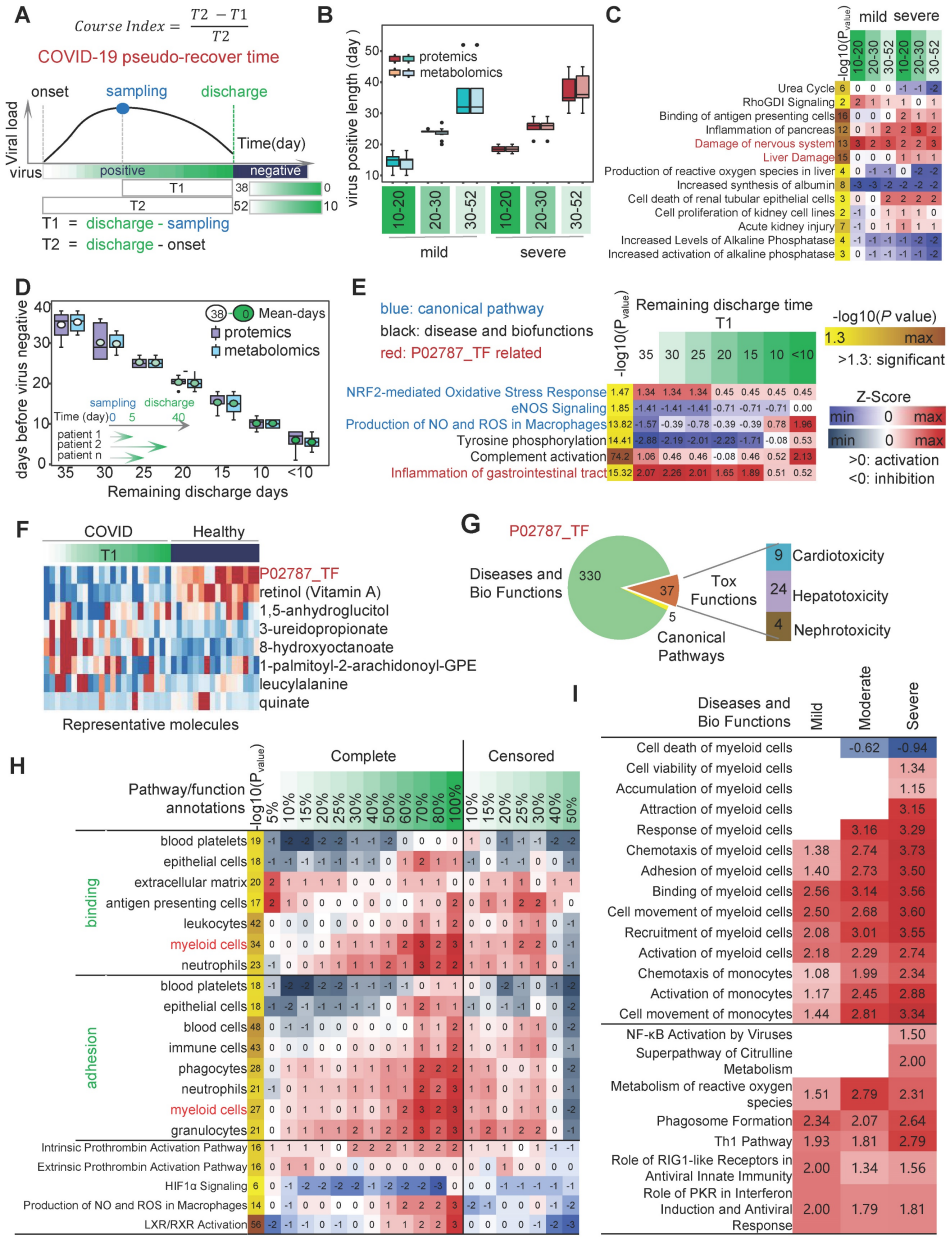
** Analysis of the dynamic changing of biological events based on pseudo-recovery timeline model. A,** Schematic diagram of course index calculation. **B**, The temporal distribution of samples in the SARS-CoV-2 "positive length" (y2 = time from onset to discharge) group. **C**, Activation of canonical pathways, disease and biofunctions in "positive length" group. **D**, The temporal distribution of samples in the SARS-CoV-2 "negative point" group. **E**, Activation of canonical pathways, disease and biofunctions in "positive length" group. **F**, Expression heatmap of 8 features related to the pseudo-recovery timeline model. **G**, Quantity of biological events in which the TF genes of the optimal features were involved. **H**, On the pseudo-recovery timeline model, the representative statistically significant biological events associated with COVID-19 progression. **I**, Exploring the impact of plasma multi-omics data on myeloid cells and other important immune phenotypes using independent test sets. The representative statistically significant biological events associated with COVID-19 progression.

When infected with SARS-CoV-2, there is a constant battle between the viral load and the immune power of the host. Disease progression (as described in the previous section) or a negative SARS-CoV-2 nucleic acid test would be two completely different outcomes of the battle. Changes in serum molecular expression profile are a landscape of COVID-19-induced disordered biological events. Especially, the changes in expression profiles could reflect the damage of cells, tissues and organs by SARS-CoV-2. Therefore, we constructed a pseudo-recovery timeline model suitable for real-world COVID-19 by correcting for individualized biological variation according to the timing of the patient's infection, different sampling points and discharge times, and calculated a course index that directly reflects the different disease stages (Figure 3A). The course index consists of two parameters, remaining discharge time T1 (range 0-38) and total disease duration T2 (range 10-52). The ability to counteract COVID-19 varies greatly from patient to patient. In this study, some patients took 10 days from onset to recovery (the shortest) and others took 52 days (the longest).

To explore the effect of disease duration on the degree of organismal damage, we assessed a combination of two indicators, including 1.) whether the patient experienced severe; and 2.) the length of T2. The sample was divided into six groups: 10-20, 20-30 and 30-52 for the mild group; 10-20, 20-30 and 30-52 for the severe group (Figure 3B). The IPA results showed that the "Urea cycle" was significantly inhibited in the severe group compared to the non-severe group 36,37. The "Urea cycle" was more inhibited in long-positive patients (T2 = 30-52) compared to short-positive patients (T2 = 10-20). There was no significant difference in the length of disease duration and disease severity in the neurological damage caused by SARS-CoV-2 compared to healthy controls. There was a significant decrease in alkaline phosphatase levels and activity in COVID-19-infected patients compared to healthy controls. Patients in the severe group had a higher degree of liver injury compared to the mild group (Figure 3C).

Next, to explore the trends of changes in biological events in the host during the COVID-19 recovery phase. We divided the samples into 7 groups according to T1 length, with mean values of 35, 30, 25, 20, 15, 10 and <10 days (Figure 3D). In the host, specifically, the longer the time until discharge, the more intense the oxidative stress response as well as the weaker the pathways or functions associated with immune signaling (immunosuppression). As COVID-19 patients recovered, the inflammatory state of the gastrointestinal tract was also alleviated (Figure 3E). We found that eight indicators such as P02787_TF, retinol (vitamin A) and leucine could reflect the progressive state of the disease in different ways (Figure 3F). Among them, P02787_TF was involved in significant activation (or inhibition) of 372 different items, including classical pathways, disease and biological functions, and toxin functions (Table S1). The abnormal expression of P02787_TF affected a variety of important functions, including "gastrointestinal inflammation", "neurological damage", and "liver damage" (Figure 3G).

Next, we applied the pST-CA strategy by aligning duration of individualized COVID-19 course, and developed pseudo-recovery timeline model based on "course index". we found that in severe patients, two prothrombin activation pathways were significantly suppressed as the disease progressed. As described in previous studies 13-15, there was a strong association between changes in platelet counts as well as concentrations of platelet aggregates and the severity, mortality, respiratory status and level of vascular endothelial dysfunction in COVID-19 patients (Figure 3H). At an overall level, fat metabolism levels of hosts and blood levels of immune cells, on a pseudo-recovery timeline model, gradually recovered (Figure 3I).

The data analysis results of an independent test set showed that a group of monocyte with a high proportion of cell numbers showed a gradually metabolic-active phenotype with increasing disease severity. In this study, we included T1 multi-omics data from Yapeng Sun et al. for activation analysis of multiple phenotypes of myeloid cells such as monocytes 33-35. The results showed that as the severity of the COVID-19 increased, the levels of chemotaxis, activation, and cell movement activation in monocytes gradually increased. Compared with healthy individuals, the "Superpathway of Citrulline Metabolism" was significantly activated in the COVID-19 severe patient group. Overall, as the severity of COVID-19 increases, metabolic related pathways such as "Production of NO and ROS in Macrophages", "Superpathway of Citrulline Metabolism", and "Metabolism of reactive oxygen specifications" are activated. At the same time, the activation of various immune cells represented by myeloid cells gradually increases (Figure 3I).

### Identifying Common Biological Events and Possible Sequelae in COVID-19-infected Patients for Potential Drugs

**Figure 4:**
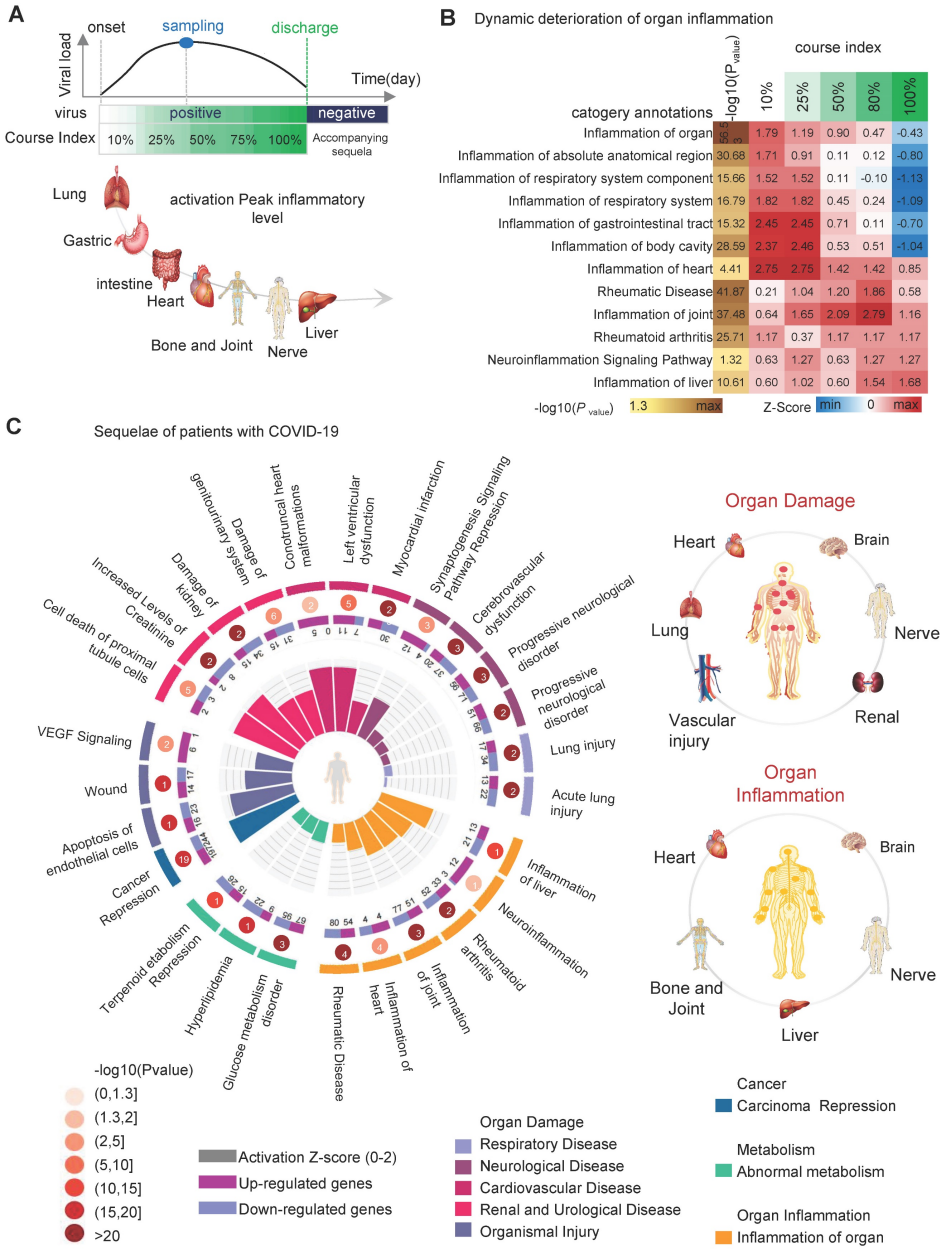
** Analysis of organ damage and sequelae. A**, the chronology of organ damage based on pseudo-recovery timeline model. **B**, the statistically significant and interested biological events associated with COVID-19 pseudo-recovery timeline model. **C**, the comprehensive map of the risk of sequelae.

When COVID-19 recovers, the typical clinical symptoms disappear. However, abnormal molecular patterns in the organism persist, causing functional abnormalities in cells, tissues, and organs. The persistent functional abnormalities lead to sequelae. IPA results based on a pseudo-recovery timeline model show a dynamic deterioration of organ inflammation in addition to the molecular and cellular levels. As the disease progresses, progressive inflammation and damage occur in the respiratory system, body cavities, gastrointestinal tract, heart, joints, nerves, and liver. The respiratory system was the earliest organ to develop inflammation and produce damage, and by the time COVID-19 patients were discharged (course index: 100%), inflammation of the respiratory system and inflammation of the gastrointestinal tract had largely disappeared. However, inflammation in the heart, bones and joints, nervous system, and liver remained localized (Figure 4A-B), showing large differences in the level of inflammation and anti-inflammatory repair capacity of different tissues and organs after viral infestation. Comprehensive analysis of the IPA results leads us to speculate that the possible sequelae of the patient after healing include organ damage (including heart, brain, lung, blood vessels, kidney and nerves), organ inflammation (including heart, brain, nerves, liver and bone and joint) and abnormal metabolism (hyperlipidemia, terpenoids, sugar, etc.) (Figure 4C, right). The abnormal biological functional signals persist after the patient heals. For example, the "Synaptogenesis Signaling Pathway" (zscore = -1.29) remained inhibited; cardiac and renal injury signals were activated, including "Left ventricular dysfunction" (zscore = 1.96), "Conotruncal heart malformations" (zscore = 1.98), "Damage of genitourinary system" (zscore = 1.34) and "Cell death of proximal tubule cells" (zscore = 1.96) (Figure 4C) (Table S2).

### Upstream regulator activity and DEPs synergies predict the drug target for SARS-CoV-2

**Figure 5:**
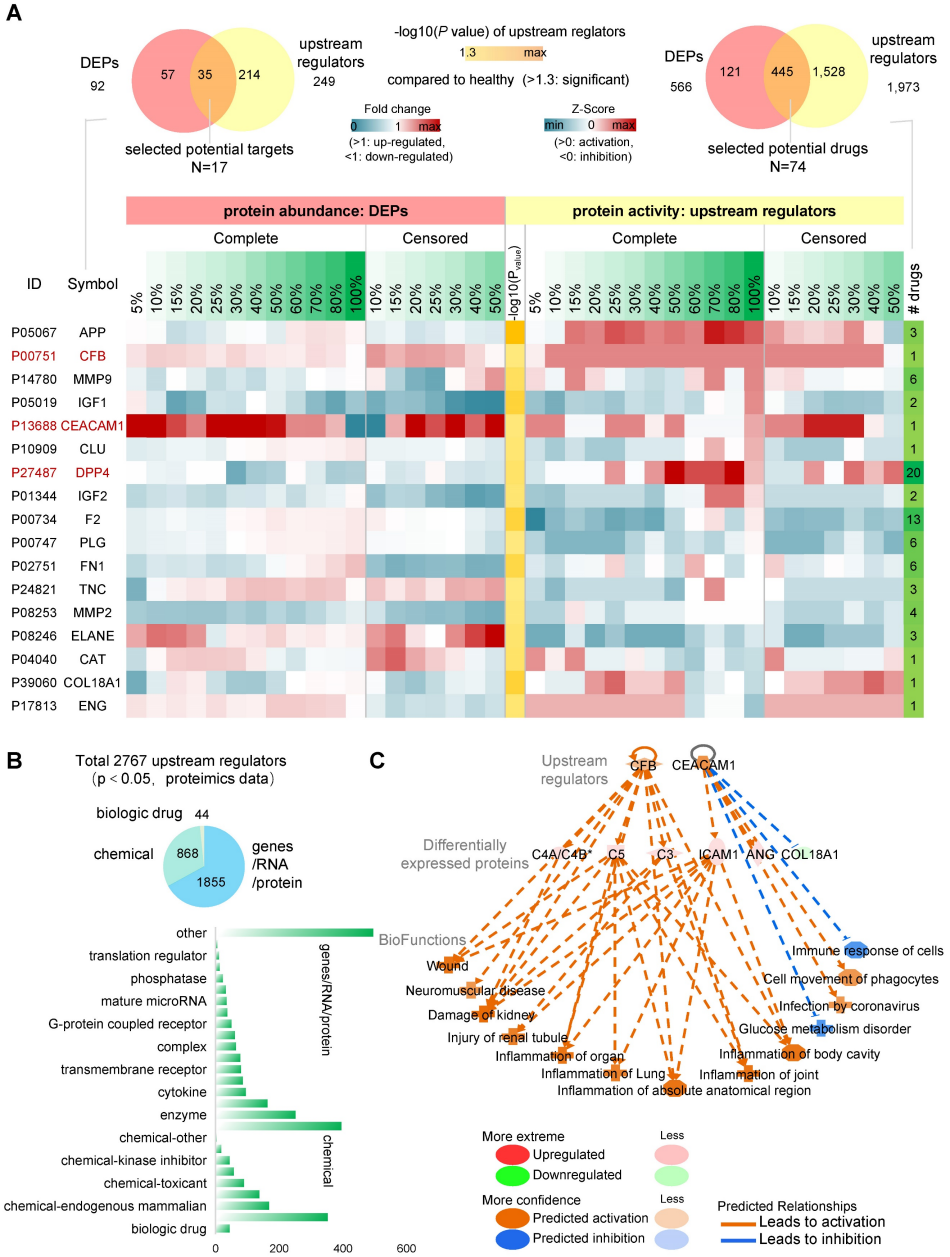
** Upstream regulator analysis of IPA platform. A**, (left top) total drug-available DEPs and upstream regulators. (right top) the number of available drugs. (bottom) Zscore heatmap of 17 protein regulators associated with pseudo-recovery timeline model and available drugs. **B**, Distribution of upstream regulating factor categories. **C**, Upstream regulation factor network prediction diagram.

The screening of potential targets with available drugs can provide an effective strategy for the body to deal with the possible sequelae. We predicted upstream regulator activity using upstream regulator analysis of IPA platform (URA) based on the COVID-19 pseudo-recovery timeline model. We compared the drug data information contained in the QIAGEN Biomedical Knowledge Base and identified 92 DEPs with specific drug targets, including 566 specific target drugs (Figure 5A, top left). URA predicted 2,767 differential upstream regulators, including gene, RNA, protein, and chemical, for 521 DEPs (Figure 5B). There were 249 upstream regulators with specific drug targets and a total of 1,973 specific targeted drugs. DEPs represent differential expression abundance (fold change, up-regulated or down-regulated), while upstream regulators represent activity (activated or suppressed). By comprehensive consideration of abundance and activity, we selected 35 kinds of candidate drug-available targets with 445 drugs. Considering the dynamic change curve of protein abundance and activity during COVID-19, we ticked out 17 high variable drug targets with 74 kinds of available drugs (Figure 5A, bottom, right).

The results of the pathway and functional analysis showed that all 17 candidate targets were involved in most of the abnormal biological events, including neurological damage, metabolic abnormalities, etc., and were associated with varieties of COVID-19-related sequelae (Table S3). Among them, CFB, CEACAM1 and DPP4 had a significantly correlated pattern of change in the pseudo-recovery timeline model, not only in abundance but also in activity. CEACAM1 and CFB remained in a relatively high expression state until recovered. In the regulator network analysis, CFB and CEACAM1 mainly affected three serum protein products, C4A/C4B*, C5 and C3, ICAM1, ANG and COL18A1, respectively (Figure 5C). Overall, these candidate drug-available protein targets are involved in different pathways of organ inflammatory response, immune response, and metabolic disorders in terms of both activities represented by upstream regulatory factors and abundance represented by differentially expressed proteins.

### CEACAM1 and ACE2 bind simultaneously to different domains of SARS-CoV-2 spike protein

**Figure 6:**
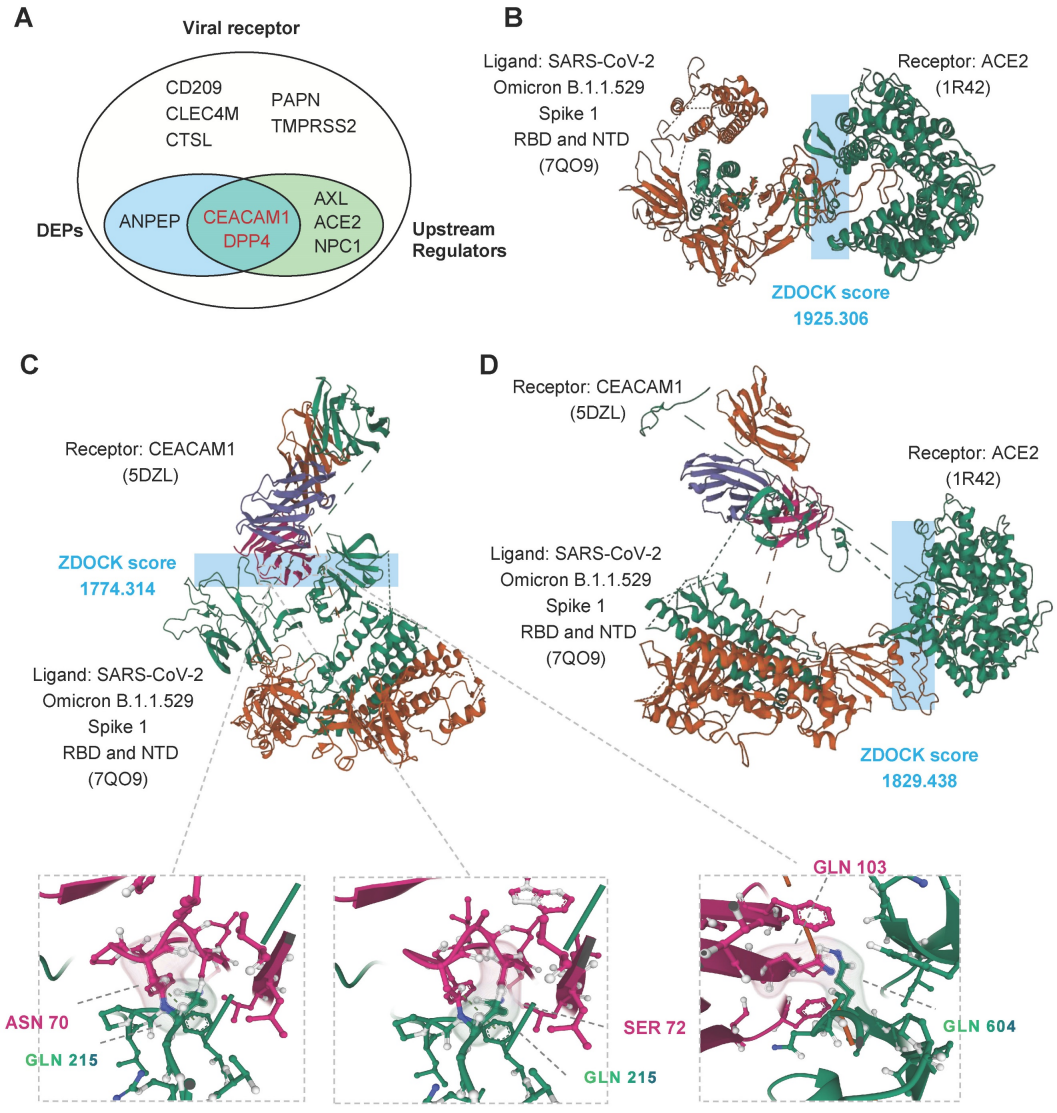
** Molecular docking simulations. A**, Venn diagram of known viral receptors intersecting with DEPs and upstream regulators. **B**, Optimal model score for binding of the S1 subunit RBD region of Omicron to ACE2. **C**, Optimal model score for binding of the S1 subunit RBD region of Omicron to CEACAM1. **D**, Optimal model score for binding of the S1 subunit RBD region of Omicron to ACE2 and CEACAM1.

Among all 11 kinds of known receptors of coronavirus 38-44, we identified three kinds of proteins with abnormal abundance (CEACAM1, DPP4 and ANPEP) and five kinds with abnormal activity (CEACAM1, DPP4, AXL, ACE2 and NPC1) (Figure 6A). The pathogenic virus of COVID-19, SARS-CoV-2, binds to the cell surface protein ACE2. The binding site is in the receptor binding domain (RBD) of the S1 subunit of the spike protein. We used ZDOCK 45 to perform molecular docking simulations on S1 (containing NTD and RBD) of the spike protein of SARS-CoV-2 omicron variant. ZDOCK results showed that the optimal model score for the binding of the S1 subunit RBD region to ACE2 was 1925.306, with a strong interaction between the two (Figure 6B).

To investigate the potential of CEACAM1 as a viral receptor involved in COVID-19 disease, we performed a molecular docking simulation analysis of SARS-CoV-2 S1 protein with CEACAM1 protein. ZDOCK results showed an optimal model scoring result of 1774.314 (Figure 6C, up) for two bindings, with similar binding capacity as ACE2 (Figure 6B). The visualization analysis showed that S1 protein could interact with residues ASN70 and SER772 of CEACAM1 through GLN215 in the NTD region, thus achieving stable binding of both (Figure 6C, bottom). Therefore, CEACAM1 and ACE2 can interact with the NTD and RBD of the S1 subunit of SARS-CoV-2 omicron variant spike protein, respectively. The S1 subunit-CEACAM1 complex was molecularly docked to ACE2. The results showed that SARS-CoV-2 bound to CEACAM1 through the S1 NTD region and then the score of the optimal model of binding to ACE2 was 1829.436. Therefore, CEACAM1 and ACE2 can act as dual receptors to bind the NTD and RBD of SARS-CoV-2 spike protein, respectively, and participate in the infiltration of SARS-CoV-2 into target cells (Figure 6D).

## Discussion

COVID-19 is a threat to all of us. In addition, the long COVID-19 and the risk of sequelae are still not well understood. Analysis of the different responses of patients with different severity, duration, and stages of SARS-CoV-2 infestation is beneficial for a systematic understanding of COVID-19 disease and for revealing the coping mechanisms of the organism. In this study, we used serum multi-omics data to identify common features among different types of COVID-19-infected patients. In contrast to previous studies, we considered that resistance to SARS-CoV-2 differs among patients. Considering individualized immune power differences, we constructed a pseudo-recovery timeline model to correct the error, which is suitable for real-world COVID-19. Severe index and course index could directly reflect the occurrence and the infection point of biological events in temporal and spatial. It comprehensively revealed the effect of severe COVID-19 before and after the severe point. In this study, we used information that includes the molecular dynamic patterns of serum abnormalities in patients and their effects on hundreds of signaling pathways, thousands of biological functions/diseases, cardiac, liver, and kidney organ toxicity, and other biological events after SARS-CoV-2 infected target cells, tissues, and organs.

After individualized differential correction, we identified severely impaired coagulation-related function in severe patients, which is highly consistent with previous findings 13-15,46. In a sequelae analysis based on a pseudo-recovery timeline model, we found that although respiratory inflammation and gastrointestinal inflammation were largely healed in the healed patients, there appeared to be irreversible damage to the heart, nervous system, and liver 47,48. The neurological inflammation and damage were present throughout the course of the patient's illness. Therefore, the findings of this study suggest that clinicians should consider whether a patient has previously been infected with SARS-CoV-2 when diagnosing or treating brain or neurological disease.

Generally, it is believed that the sequelae of COVID-19 occur mostly in severe patients. Our data suggest that even in mild patients, SARS-CoV-2 infection can still cause abnormal molecular patterns in the body, and some of these abnormalities can gradually resolve with the onset, exacerbation and gradual recovery of the infection, such as "Inflammation of respiratory system", "Inflammation of gastrointestinal tract", etc. "Inflammation of the gastrointestinal tract", etc. Some abnormalities persist, such as "Neuroinflammation Signaling Pathway", "Rheumatoid arthritis" and "Inflammation of liver". Inflammation of liver", etc. When patients with mild disease recover, molecular patterns including organ damage, inflammation, and metabolic abnormalities are still present in the body. For example, neurological damage related molecular patterns such as "Synaptogenesis Signaling Pathway" (zscore = -1.29) and "Neuroinflammation Signaling Pathway" may be associated with the development of sequelae such as thought disorders, depression or anxiety.

We further included multi-center data for analysis, which showed that patients discharged at home (even those claiming no sequelae) had neuroinflammatory damage signals in their blood that remained activated (z-score > 0) up to 170 days after the onset of COVID-19. Therefore, the effect of SARS-CoV-2 infection on the organism cannot be ignored, even in mildly COVID-19 patients. It still has negative, long-term and subtle effects on the nervous system, etc.

In addition to organ inflammation and damage, SARS-CoV-2 invasion activates the anti-cancer effect of the organism in general. When combined with PD-L1 therapy after viral infection, patients are stimulated to produce a stronger immune response, which effectively destroys tumor cells. However, in patients with severe COVID-19 infection, the ability of immune cells to kill tumors in order to destroy the exogenous virus was significantly diminished. Research suggests that increased immune checkpoint receptors are associated with T-cell exhaustion in severe COVID-19 49. After SARS-CoV-2 infection, a cytokine storm induces T cell hyperactivation and ultimately accelerates cellular death 50. In severe COVID-19, CD8 T-cells decrease the cell-mediated immune response to the virus while upregulating immunosuppressive markers such as PD-1 and mucin-3 51,52. Therefore, more research is needed to determine whether detoxified COVID-19 viruses will be beneficial in cancer therapy.

In this study, we found that the multi-omics data of patients' blood after SARS-CoV-2 infestation of the organism can synthetically reflect the activation profiles of abnormal biological events, i.e. molecular symptoms, that are occurring and about to occur in the patient's organism. With the exception of death and isolated cases of recurrent disease, COVID-19 generally undergoes two phases of deterioration and improvement from the onset to recovery. The overall WHO classification also shows a low-high-low transition trend as the disease progresses. The generation of sequelae provides macroscopic evidence that patient discharge from the hospital does not mean that the organism returns to its pre-infection health status. On the contrary, even if the WHO classification is the same (macroscopic clinical symptoms are similar), the organism in deterioration and in recovery presents a completely different activation landscape of abnormal biological events within the organism (microscopic molecular symptoms are different). Pseudo-recovery timeline model can effectively reveal abnormal molecular phenotypes in different disease course groups at the same severity. The pST-CA strategy has an important revelatory value for analysis of the progression of various diseases.

Disease regression of COVID-19 is closely related to the abnormal phenotype of immune cells. The results of this study showed that blood platelets, antigen presenting cells, leukocytes, myeloid cells and neutrophils showed different dynamic activation trends as the disease progression (figure 3H). Further studies on long COVID-19 showed that abnormal patterns of immune cell activation persisted in the blood of patients during the recovery period and after discharge home (follow-up sampling to 170 days post-onset). For example, "Activation of natural killer cells", "Crosstalk between Dendritic Cells and Natural Killer Cells ", "T Cell Exhaustion Signaling Pathway" and "T cell response" (Figure S2).

Adaptive evolution of phenotype-associated dominant cell subpopulations is an important strategy for immune cells to help the organism fight against external pathogenic microbial invasion. Single-cell sequencing is a powerful weapon to reveal the heterogeneity of immune cells. Several single-cell transcriptome sequencing-based studies have revealed immune cells closely related to COVID-19 and their characteristic subtypes, including "adaptive NK cells”, NK cells with a distinctive early IFN-α profile, and KIR^+^CD8^+^ T cells, etc. 53-55. It will be one of the important directions in the future to map the activation of abnormal biological events of single cell subpopulations under pseudo-recovery timeline model and further reveal the specific targets of abnormal phenotype-related single cell subpopulations.

The establishment of the pseudo-recovery timeline model provides researchers with more possibilities to further approach the real scenario of the organism against pathogenic microorganisms. In the war scene, the enemy and the defender will appear together. Whether the immune cell subpopulations that develop dynamically along with the pseudo-recovery timeline model are the drivers or the enemies of disease regression needs more support in terms of analysis strategies and algorithm development. This will likewise be an important issue to be addressed by precision medicine in the future.

In summary, this study used serum multi-omics data from COVID-19-infected patients to systematically map the dynamics of biological events at various levels in the organism at various stages after SARS-CoV-2 infection in COVID-19 patients. Combining information on the abundance represented by differential proteins and protein activity represented by differential upstream regulatory factors, a novel perspective was taken to find drugs that can counteract COVID-19 at dynamic levels across the disease course.

## Supplementary Material

Supplementary figures.

Supplementary table 1 - TF related pathway function and ToxFunctions.

Supplementary table 2 - sequelae related biofunction.

Supplementary table 3 - target related biofunction.

## Abbreviations

GSEA: Gene Set Enrichment Analysis
IPA: Ingenuity Pathway Analysis
COVID-19: 2019 coronavirus disease
SARS-CoV-2: severe acute respiratory syndrome coronavirus 2
CNS: central nervous system
TME: tumor microenvironment
NO: near onset group
BS: before-severe point group
SP: severe point group
AP: after-severe point group
PS: persistent severe group
