# Supplementary material for: The landscape of abnormal pathway activation confers COVID-19 patients' molecular sequelae earlier than clinical phenotype
Source: Theranostics. 2023 Jun 12;13(10):3451–66. doi: 10.7150/thno.83405 (PMC10283057; doi:10.7150/thno.83405)
Supplement: Supplementary file 1 — Supplementary figures. [file thnov13p3451s1.pdf]

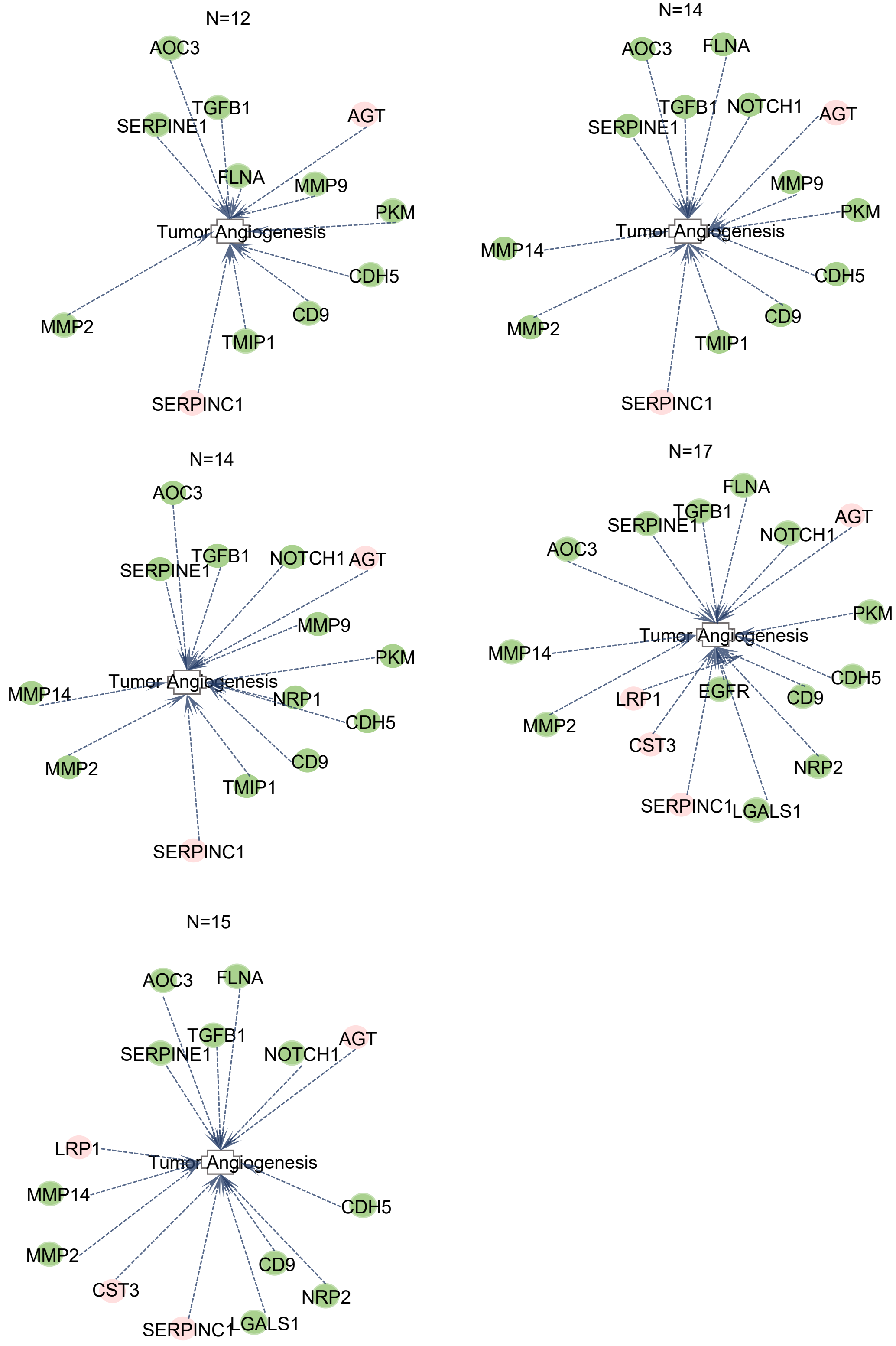

N=12
AOC3
TGFB1
SERPINE1
AGT
FLNA
MMP9
PKM
Tumor Angiogenesis
CDH5
CD9
MMP2
TMIP1
SERPINC1
N=14
AOC3
FLNA
TGFB1
NOTCH1
AGT
SERPINE1
MMP9
PKM
MMP14
Tumor Angiogenesis
CDH5
CD9
MMP2
TMIP1
SERPINC1
N=14
AOC3
SERPINE1
TGFB1
NOTCH1
AGT
MMP9
PKM
MMP14
Tumor Angiogenesis
NRP1
CDH5
CD9
MMP2
TMIP1
SERPINC1
N=17
FLNA
TGFB1
SERPINE1
AGT
NOTCH1
AOC3
PKM
MMP14
Tumor Angiogenesis
CDH5
LRP1
EGFR
CD9
MMP2
CST3
NRP2
SERPINC1
LGALS1
N=15
AOC3
FLNA
TGFB1
SERPINE1
NOTCH1
AGT
LRP1
Tumor Angiogenesis
MMP14
CDH5
MMP2
CD9
CST3
NRP2
SERPINC1
LGALS1

Supplementary Figure S1| **Changing patterns of tumor angiogenesis inhibition. A**, NO phase, genes involved in inhibiting the tumor angiogenesis pathway. **B**, BS phase, genes involved in inhibiting the tumor angiogenesis pathway. **C**, SP phase, genes involved in inhibiting the tumor angiogenesis pathway. **D**, AS phase, genes involved in inhibiting the tumor angiogenesis pathway. E, PS phase, genes involved in inhibiting the tumor angiogenesis pathway.

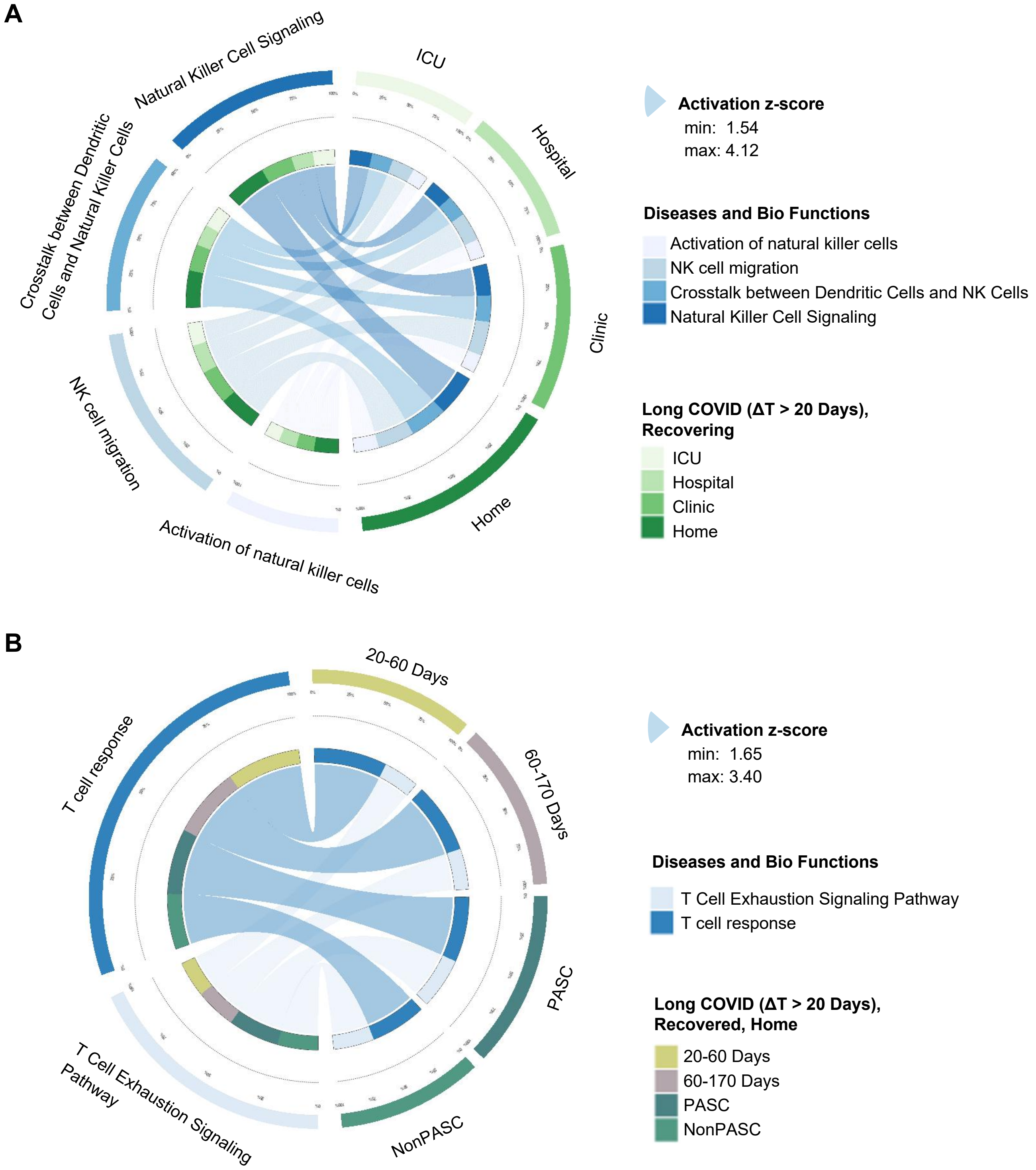

A
Natural Killer Cell Signaling
ICU
Hospital
Clinic
Home
Activation of natural killer cells
NK cell migration
Crosstalk between Dendritic Cells and Natural Killer Cells
Activation z-score
min: 1.54
max: 4.12
Diseases and Bio Functions
Activation of natural killer cells
NK cell migration
Crosstalk between Dendritic Cells and NK Cells
Natural Killer Cell Signaling
Long COVID (ΔT > 20 Days), Recovering
ICU
Hospital
Clinic
Home
B
20-60 Days
60-170 Days
PASC
NonPASC
T Cell Exhaustion Signaling Pathway
T cell response
Activation z-score
min: 1.65
max: 3.40
Diseases and Bio Functions
T Cell Exhaustion Signaling Pathway
T cell response
Long COVID (ΔT > 20 Days), Recovered, Home
20-60 Days
60-170 Days
PASC
NonPASC

Supplementary Figure S2| **Circos plot shows the representative abnormal activation phenotype of NK and T cells in different groups.** **A**, Different activation z-score of NK cell related Diseases and Bio Functions in four severity states during the recovery period presented by circos plots.The circos plot represented activation z-score values of each Functions. All z-scores are greater than 0. The minimum value is 1.54, and the maximum value is 4.12. **B**, The representative abnormal activation phenotype of T cells in different groups after the recovery of patients with long COVID-19. The circos plot represented activation z-score values of each Functions. All z-scores are greater than 0. The minimum value is 1.65, and the maximum value is 3.40.
